# Mr. Cuming, on the Influenza

**Published:** 1803-10-01

**Authors:** Ralph Cuming

**Affiliations:** Romsey, Hants


### *To the Editors of the Medical and Phyſical Journal.*

GENTLEMEN,

AFTER a thorough investigation of the opinions adduced, respecting the disease, *entitled* Influenza; when a majority amounting to nine-tenths of the medical world are agreed with respect to its non-contagious nature, all controversy should now cease, as it is certain that the proof, though not positive, is sufficiently presumptive. As there has been diversity of opinion concerning its nature, so have there been different rules laid down for its cure. Some condemn the use of the lancet, asserting that phlebotomy is extremely pernicious, whilst others declare that a contrary practice has been, in certain instances, fraught with the happiest effects.

It fell to my lot, from having been appointed to attend the paupers of the outer and inner parish of Romsey, to see much of the prevailing epidemic; and if the following observations should tend to elucidate the subject, my trouble in drawing up this statement will be amply compensated.

I am

I am of opinion that the remote causes of this disease originated in the sudden change of atmosphere, which we experienced in this county; a change, I believe, generally felt throughout the United Kingdoms, as well as upon the Continent. As I kept no meteorological diary, the facts relative to the weather are deduced from my memory. The heat during the greatest part of the days in the latter end of March and beginning of April, was such that few people ever before endured at that season; its approach was as sudden as its departure, not giving the constitution an opportunity of accommodating itself, either to the expansion occasioned by the heat, or the stricture produced by the cold. If the above plain statement be correct, we may save ourselves the trouble of ransacking the bowels of the earth, or of darting with microscopic eye into the insect kingdom, to discover what seems to have perplexed our great philosophers.

I myself had the prevailing epidemic, which I attributed to taking too much exercise during the warm unseasonable weather, and not to contagion; for had the complaint been infectious, Mrs. C. and the rest of my family would surely have suffered from it. I was very ill four or five days, but not so as to prevent me from attending my patients. I was first seized with considerable pain of my head, a sense of languor and debility, heaviness of my eyes; at times I felt a sense of chilliness. I here wish to observe, that I conceive this complaint to be very different from catarrh, for neither in myself nor any of my patients, did I observe any affection of the mucus membrane of the nose, or watery eye, so commonly noticed; by taking a calomel purge, using pediluvium, and washing down a bolus pulv. antim. gr. v. opii gr. i. with white wine whey, and by perseverance in the bolus and whey for three nights, I found myself in a convalescent state; but my head for a fortnight or more, on the slightest exertion, was subject to pain and throbbing, something like palpitation, which I attributed to a state of atony in the system, and consequent increased vascular action. By way of divesting my brethren of prejudice with respect to bleeding in this disease, I have to observe, that the case of a patient, 61 years of age, ranked with the worst that came under my care. This old man was severely affected with the prevailing epidemic, and I have reason to think, if the dictates of timidity had influenced my conduct, he must either have died, or lingered out the remainder of his days in that distressing malady phthisis pulmonalis. He was at first taken with all the usual usual symptoms in an aggravated degree ; the pyrexia, languor and debility were great, dyspnœa and painful inspiration intolerable ; he complained of a stitch and tightness across his chest, so that, to use his own expression, there was not room for the air to descend into his lungs. His pulse though frequent, was small and tremulous, his debility such that with difficulty he could raise his head from the pillow. The treatment of such a case required time and consideration, yet there was not a moment to be lost; something was to be done, though not rashly, for without relief death appeared certain. The candid reader will easily conceive the difficulty and dilemma attendant on such a case; his debilitated state, his tremulous, quick, and weak pulse, according to some people's theories, forbade the use of the lancet, particularly in this disease, yet I bled him, and to the amount of ten ounces; though the blood did not flow from a large orifice, or quickly, yet I never in the whole course of my practice beheld so much coagulable lymph as presented itself to my view the next day; it was truly a buffy coat, not many degrees removed from the nature of a bull's hide, and considerably thicker; it constituted a third part of the crassamentum covering its whole surface. The patient's bowels being constipated, I deemed it necessary to give him a calomel purge, which operated very freely, yet after these evacuations the stitch and tightness still continued, and in less than twenty-four hours I repeated the bleeding to the same amount, and blistered him; the buffy coat did not occupy so large a space, nor was it near so thick. By observing the antiphlogistic plan, by giving small and repeated doses of soda nitrata, with the addition of a small quantity of antim. tartar. the violence of his complaint began to subside ; after which, by the administration of gentle anodynes conjoined with tinct. scillæ, he began to expectorate, and his recovery after his pulmonic complaints ceased, was expedited by the exhibition of tonics. Two other cases of a similar nature yielded to the same plan of treatment; one of them a poor hard working man, from the violence of his pectoral complaints, nearly fell a victim to phthisis pulmonalis ; for several days this patient's expectoration was so excessive, that he filled a pint pot with mucus of a purulent appearance, though I did not subject it to the test of experiment, knowing that common tests are as uncertain as they are fallacious ; he had also colliquative night sweats, frequent and small pulse, and a constant pain in the head. I here had an opportunity of trying the effects of Dr. Magennis's favourite remedy medy, tinct. digitalis saturata, beginning with small doses, and increasing them gradatim; and I must candidly confess it appeared to succeed and answer my most earnest expectation; the sweating gradually left him, the expectoration began to diminish, and the cure was completed by means of decoct. cinch. flavæ cum acido vitriolico, q. s. ad gratam aciditatem. The success of this practice, unadorned with any fluctuating theory of this epidemic with the fine name of *influenza*, is sufficient to enable me to assert that it is by no means a fatal disease, for I did not lose a patient notwithstanding I bled; yet I do not wish it to go abroad, that because I adduce an instance or two of the success of this practice, that I conceive it to be always necessary; by no means, for I would have quite the reverse to be understood, as I met with many cases that would not bear bleeding; and I must observe, that much must ever be left to the judgment of the practitioner, and that his opinion should not always yield to the dogmatical dictates of medical authors, of whom I think Cullen, in point of *good sense, candour, modesty*, and practical knowledge, deservedly holds the pre-eminence. I am afraid I have trespassed too much on the limits of your excellent publication, for in works of this kind people should consider that by being too diffuse on some subjects, others, as worthy and instructive, may be lost to the public: Yet I cannot close this essay, without animadverting on Dr. Magennis's account relative to the plan of treatment adopted by a naval surgeon in a ship of war; his observations may be ill timed, nay even unjust, for the censure which they seem to convey may be unmerited. What necessity was there for having mentioned the name of the ship? The characters of men in public situations should be delicately handled; and extremely glad shall I be, if the statement of my cases should tend to exculpate the surgeon, or wipe off any stain that may attach to his character: for it is evident, whether censure was intended or not, that the world does not always construe things in the most charitable way. I have no desire to wean gentlemen from their own opinion, *sed quod vidi ad scripsi.*

*Romsey, Hants,*

*August* 10, 1803.

I am, &c.

RALPH CUMING.

*A* Case

